# Impact of flu on hospital admissions during 4 flu seasons in Spain, 2000–2004

**DOI:** 10.1186/1471-2458-7-197

**Published:** 2007-08-08

**Authors:** Annick D Lenglet, Victoria Hernando, Pilar Rodrigo, Amparo Larrauri, Juan DM Donado, Salvador de Mateo

**Affiliations:** 1 European Programme for Intervention Epidemiology Training (EPIET), Smittskyddinstitutet/EPI, Nobels Vag 18, 17182, Solna, Sweden; 2 Programa de Epidemiología Aplicada de Campo (PEAC), National Centre for Epidemiology, Public Health Institute Carlos III, C/Sinesio Delgado, 6 28029 Madrid, Spain; 3 Epidemiological Surveillance, National Centre for Epidemiology, Public Health Institute Carlos III, C/Sinesio Delgado, 6 28029 Madrid, Spain

## Abstract

**Background:**

Seasonal flu epidemics in the European region cause high numbers of cases and deaths. Flu-associated mortality has been estimated but morbidity studies are necessary to understand the burden of disease in the population. Our objective was to estimate the excess hospital admissions in Spain of diseases associated with influenza during four epidemic influenza periods (2000 – 2004).

**Methods:**

Hospital discharge registers containing pneumonia, chronic bronchitis, heart failure and flu from all public hospitals in Spain were reviewed for the years 2000 to 2004. Epidemic periods were defined by data from the Sentinel Surveillance System. Excess hospitalisations were calculated as the difference between the average number of weekly hospitalisations/100,000 in epidemic and non-epidemic periods. Flu epidemics were defined for seasons 2001/2002, 2002/2003, 2003/2004.

**Results:**

A(H3N2) was the dominant circulating serotype in 2001/2002 and 2003/2004. Negligible excess hospitalisations were observed during the 2002/2003 epidemic where A(H1N1) was circulating. During 2000/2001, flu activity remained below threshold levels and therefore no epidemic period was defined. In two epidemic periods studied a delay between the peak of the influenza epidemic and the peak of hospitalisations was observed. During flu epidemics with A(H3N2), excess hospitalisations were higher in men and in persons <5 and >64 years higher than 10 per 100,000. Pneumonia accounted for 70% of all flu associated hospitalisations followed by chronic bronchitis. No excess flu-specific hospitalisations were recorded during all seasons.

**Conclusion:**

Flu epidemics have an impact on hospital morbidity in Spain. Further studies that include other variables, such as temperature and humidity, are necessary and will deepen our understanding of the role of each factor during flu epidemics and their relation with morbidity.

## Background

Influenza virus causes an acute disease of the respiratory pathways, with high potential for person to person transmission. The World Health Organization (WHO) estimates that there are approximately 3 to 5 million cases and 250,000–500,000 deaths due to flu each year worldwide [1]. Flu epidemics in the European Region occur annually during the winter season causing a high number of cases and deaths.

With the introduction of the flu vaccine the number of influenza cases and deaths have decreased significantly, especially in vulnerable groups [2,3]. In Spain, the flu vaccine was introduced in the early 1980s. Since then studies have showed that flu vaccination prevents hospital admissions for pneumonia in persons older than 65 years (OR = 0.21, CI95% = 0.09–0.55) [4] and is 22% effective in preventing mortality in persons over 75 with chronic disease [5]. The Ministry of Health vaccination guidelines from 1992 recommend flu vaccinations to persons above 65 years of age, persons younger than 65 years who for medical reasons have a higher risk of developing flu complications, their caretakers and healthcare staff. Between 1993 and 2001, the vaccination coverage in persons with chronic medical conditions, was estimated to be 31.2% for those younger than 65 years and 46.0% for persons older than 65 years [6]. A recent study estimated that 18 million persons, 41% of the Spanish population, should receive influenza vaccination in the 2006–2007 season according to the Spanish guidelines[7].

Despite the availability of the flu vaccine, flu and the diseases associated with it continue to be an important public health issue. Flu-associated mortality has been estimated in several studies elsewhere [8-12]. However, this mortality does not represent the actual burden of the disease in the population as only a small proportion of severe flu cases are fatal [8,13,14]. Estimating the number of influenza associated cases better reflects better the burden of disease in the population. Recent studies have shown that influenza-associated hospital based morbidity in the United States, Canada, England and Wales and Hong Kong, is higher in children younger than 2 years and in persons older than 65 years [10,15-20]. A study in the Spanish Basque Country, showed that hospitalisation rates in children under 5 years were higher during influenza epidemics [21]. The impact on hospital admissions has not yet been measured for the rest of the country or in other age groups.

Our objective was to estimate the excess hospital admissions in Spain of diseases associated with influenza, during four epidemic influenza periods by gender, vulnerable groups and according to the circulating dominant influenza serotype in order to improve the existing prevention and vaccination programmes and the planning of healthcare resources.

## Methods

### Hospitalisation

We defined all-cause flu-associated hospitalisation as a patient who was discharged from hospital with pneumonia, chronic bronchitis (CB), heart failure (HF) and flu present in at least one of the 10 possible diagnostic fields. These diseases were selected as they are most related to flu-associated morbidity based in existing bibliography. We included all diagnostic fields so as to ensure the capture of all hospitalisations for diseases possibly associated with influenza.

Hospital discharge registers, also know as the Minimum Basic Set of Data (MBSD), were reviewed from all public hospitals for the years 2000 to 2004. For pneumonia we included ICD9-CM codes 480–486, which correspond to pneumonias due to all viral, bacterial and unknown causes. The ICD9-CM codes for CB included non-specific bronchitis, chronic bronchitis and emphysema (ICD9-CM: 490–492). For HF we selected ICD9-CM codes corresponding to congestive heart failure, non-specific heart failure and other specific and non-specific chronic cardio-pulmonary conditions (ICD9-CM: 428.0, 428.9, 416.8 y 416.9). For flu we used influenza: ICD9-CM code 487, which is influenza.

### Definition of epidemic and non-epidemic flu periods

Information on the circulation of flu is collected each year between epidemiological weeks 40 and 20 by the Spanish Influenza Sentinel Surveillance System (SISS) [22]. Briefly, the surveillance system uses sentinel general practitioners and paediatricians (538 during last flu season), who notify weekly the number of consultations for influenza-like illness (ILI) in their reference populations. In addition, general practitioners regularly send samples for virological confirmation to the sentinel laboratories in their region. This allows the genetic and antigenic characterisation of the influenza virus' circulating during each surveillance period. Each week, a global population adjusted influenza rate for Spain is calculated to follow the evolution of influenza activity. The sentinel surveillance system for influenza covers approximately 75% of the population nationwide.

Threshold incidence rates for epidemic influenza activity are defined for each surveillance season, using the mean incidence rates from the previous 5 years of surveillance. The viral isolation rate is estimated as the percentage of positive viral isolations out of the total number of respiratory samples sent to the laboratory.

We defined an 'epidemic' period as the interval of weeks between epidemiological weeks 40 and 20 of the winter season with an incidence rate for flu over passing the threshold for that flu season and a viral isolation rate higher than 30%. The non-epidemic period was defined as the interval of weeks between epidemiological weeks 40 and 20 not corresponding to the epidemic period.

### Analysis

To study the evolution of the influenza epidemics and hospitalisation rates during influenza surveillance seasons, we compared the ILI incidence rates per week and the weekly hospitalisation rates (number of hospital admissions/week/population) for all flu-associated diseases.

We calculated average weekly hospital admissions per 100,000 population for the epidemic and non-epidemic period of the flu season separately. We defined the excess hospital disease burden as the difference between weekly hospital admissions per 100,000 inhabitants for the epidemic and the non-epidemic period. Hospitalisation rates were calculated during each period for all-cause, pneumonia, CB, HF and flu and for the overall population, by sex and age group (under 5 years and over 64 years). Confidence intervals of 95% were calculated for all weekly admission rates.

Population figures for age and sex were obtained from the Spanish National Institute of Statistics. We used the population for the second year of a flu season as the denominator. For example, for the 2000/2001 flu season (S1), the population data for the year 2001 were used.

This study required no ethical approval as the data used for the study was obtained from anonymous data from hospital discharge registers and the influenza surveillance system with no personal identifier codes or information available.

## Results

### Epidemic periods and circulating serotypes

**Figure 1:**
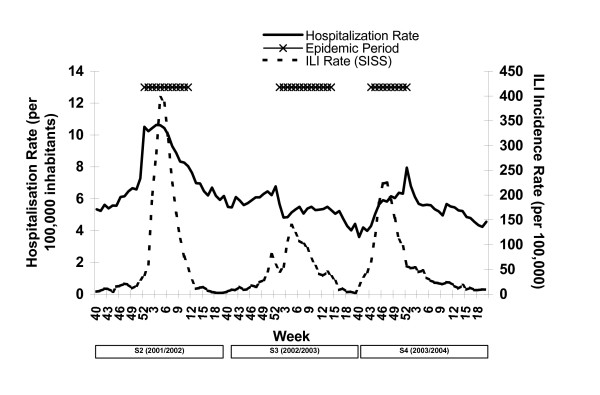
Hospitalisation rates for all flu associated diseases and ILI incidence rates (both per 100,000 inhabitants) per epidemiological week in Spain during influenza seasons 2001/2002, 2002/2003 and 2003/2004.

In S1 no epidemic flu period could be defined as the maximum weekly ILI incidence (37.6 cases per 100,000) was below the epidemic threshold. The highest weekly flu incidence recorded for the four seasons was during 2001/2002 (S2), when incidence rates reached 312.2 cases per 100,000, followed by 2003/2004 (S4) with 225.0 cases per 100000 and finally 2002/2003 (S3) with 139.3 cases per 100000 (Figure 1). The duration of the epidemic period was 12 weeks in S2, 14 weeks in S3 and 10 weeks in S4 (Figure 1).

Influenza B was the dominant circulating serotype during the S1 season. In S3 both influenza B and A(H1N1) were circulating with influenza B as the dominant serotype. Influenza A(H3N2) was the dominant serotype detected circulating in both the S2 and S4 seasons (Additional file 1).

### All-cause influenza-associated hospital admissions and ILI rates

From week 40 to 20 hospital admission rates for all-cause flu-associated disease were 247 (100,102 cases), 180 (73,358 cases) and 177 (72,481 cases) per 100.000 inhabitants in S2, S3 and S4 respectively (Figure 1). In S2 a peak was reached for all cause hospitalisations between weeks 52 and 5; and for ILI incidence between weeks 4 and 6. In S3 hospitalisations remained constant and ILI incidence peaked in weeks 4 and 5. In S4 the peak in hospitalisations occurred in weeks 52 and 1 and ILI incidence peaked in week 47.

### All age excess hospital admissions

**Figure 2:**
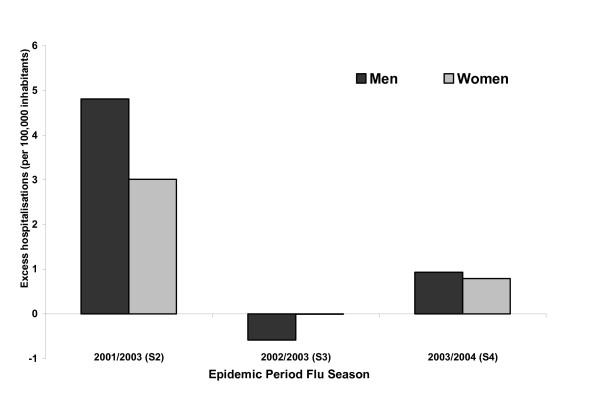
Excess average weekly hospitalisations in men and women for all-cause hospitalisations per 100,000 during influenza epidemics 2001/2002 (S2), 2002/2003 (S3) and 2003/2004 (S4) in Spain.

For the defined epidemic periods in S2 and S4 there was an excess in all cause flu-associated hospitalisations. In S3 hospital admissions in both the defined epidemic and non-epidemic periods were similar (Additional file 1). During S2 and S4 the excess hospitalisations per 100, 000 were higher in men compared to women (Figure 2); 4.81 in men and 3.01 in women in S2 and 0.93 in men and 0.79 in women in S4. In S3 no excess all-cause hospitalisations were recorded during the epidemic period for men and women.

A total of 120,407, 88,618 and 88,497 flu-associated diseases were registered for S2, S3, S4. Amongst a total of 209,698 pneumonia diagnoses, 91% were registered as the principal or secondary field of the MBSD (i.e. reason for hospital admission). The number of hospital admissions reported as principal or secondary diagnosis was 48,857 (62%) for CB and 37,389 (35%) for overall for all three seasons. Among the 1578 reported flu cases 88% were reported as primary or secondary diagnostic in the MBSD.

Pneumonia was the most common of all flu-associated diseases. In S2, 79,836 (66%) of all flu-associated diagnoses included in this study were due to pneumonia, regardless of epidemic period. This proportion was higher in S3 and the S4 season where pneumonia accounted for 63,644 (71%) and 66,218 (75%) hospital diagnoses respectively. In all three epidemic periods, hospital admissions for pneumonia were always in excess, with the highest for S2, followed by S4 and S3 (Additional file 1).

CB hospitalisations were the second most common flu-associated disease during the seasons studied, 22,892 (19%), 13,913 (16%) and 12,052 (14%) for S2, S3 and S4 respectively. The maximum number of hospitalisations for CB occurred during the epidemic period of S2 with 2.2 cases per 100,000 (Additional file 1). During S3, a negative excess morbidity was calculated. In S4 excess hospitalisations were less than 1 case per 100,000.

Hospitalisations with HF diagnosis represented 14% (n = 17323), 12% (n = 10655) and 10% (n = 9411) of all flu associated diagnoses for S2, S3 and S4 respectively. Excess HF hospital morbidity was recorded for the S2 and S4 seasons, with similar rates in both (Additional file 1). For the S3 season there was no excess in overall hospital admissions for HF.

No excess morbidity for flu specific hospitalisations was recorded in S2, S3 or S4 (Additional file 1).

### Persons below 5 years and over 64 years

The two age groups with highest number of flu-associated hospitalisations regardless of epidemic period were children under 5 years and persons above 64 years of age. In these age groups, excess hospital burden is the highest. Excess all-cause hospitalisations during epidemic periods exceed 10 cases per 100,000 in both age groups during S2 and S4 (Additional file 2). This excess is negligible during S3.

In children under 5 years, pneumonia was the principal reason for excess admissions during the flu epidemic in both S2 and S4, accounting for 98% and 99% of all excess all-cause hospital morbidity calculated for these seasons (Additional file 2). Excess hospitalisations for CB, HF and flu were below 1 per 100,000 during all three seasons in this age group.

For persons above 65 years the highest excess hospital burden was for pneumonia during all three seasons (Additional file 2). During S3 this excess was 87% and 89% lower than in S2 and S4 respectively. HF and CB had similar excess hospitalisations during all three seasons, being negligible during S3. For flu, excess hospital morbidity remained below 1 case per 100,000 during epidemic periods for all three seasons.

## Discussion

Our results suggest that during the studied influenza seasons there was an impact of flu epidemics on hospital admissions in Spain. As has been described in other studies [8,16,17], the impact of flu appears to be greater when A(H3N2) is the dominating circulating serotype of the virus, which is similar to our results from the epidemic periods of S2 and S4. This suggests that A(H3N2) is a more virulent serotype, thereby causing more severe clinical manifestations leading to hospitalisation. In our study pneumonia was the main flu-associated disease causing hospitalisations and persons below 5 years and over 64 years of age were the most vulnerable for hospitalisation during flu epidemics, as has been shown in previous studies [10,16-21].

In two of three flu epidemics studied there is a delay observed between the peak of the influenza epidemics and the peak in all-cause-flu-associated hospitalisations. One possible explanation of the delay would be that underlying medical conditions do not deteriorate immediately in persons infected with influenza. The time between infection and need for hospitalisation could reflect the delay seen in S3 and S4.

In our study we may have overestimated the excess disease burden in hospitals. Firstly, by including ten diagnostic fields from the hospital discharge registers, we could be overestimating the number of hospitalisations associated with flu. However, we have observed that a high proportion of the included flu-associated diseases were reported as principal or secondary diagnosis, reflecting that these clinical manifestations were likely to be related to the actual reason for hospital admission. Secondly, hospital discharge registers do not include laboratory information (i.e. influenza confirmation), limiting our capacity to estimate the real number of hospitalisations from influenza infection. Part of the hospitalisations that we have considered to be flu-associated might in fact be due to parainfluenza virus, respiratory synctical virus (RSV) or adenovirus [10,23]. It is likely that different respiratory virus' have different effects in different age groups. As was found by Mullooly et al., influenza infection in persons above 65 years of age and with underlying chronic pathologies was a stronger risk factor for hospitalisations for cardiac reasons than RSV infection [24]. In contrast, children under 5 months of age showed the highest rates for emergency department visits for respiratory infections in winter months due to RSV, whereas children between 6 and 23 months reported emergency department visits for influenza related infections [25].

The overestimation of the impact of flu on hospital admissions is especially a limitation if one wants to calculate in economical terms the burden of influenza in hospitals [26]. Barker estimated that the total cost associated with excess hospitalisations for pneumonia and influenza during an influenza A epidemic was equal to $300 million dollars in the United States [8]. Though prices for hospitalisation and size of population in Spain are different, costs associated with excess hospital burden from influenza associated pathologies could be substantial.

The MBSD does not include information on re-admissions, limiting the identification of duplicate admissions for the same disease in the same patient and therefore a possible overestimation of hospitalisations. However, as this limitation is present in all three seasons studied the results obtained are still comparable between them. The hospital discharge files also do not collect information about underlying risk factors that can contribute to more severe manifestations following influenza infection, such as cardio-vascular, kidney and metabolic diseases and anaemia [27].

Also, no information on flu vaccination history is available and we have not been able to evaluate the role that vaccination has in flu-associated hospital admissions during epidemic flu periods. The analysis of National Health Surveys conducted in 2001 and 2003 show that average influenza vaccine coverage in Spain in persons under 65 years of age with chronic conditions (diabetes, asthma, chronic bronchitis and heart disease) was 29.8% and 35.3% respectively [28]. In persons above 65 years of age the average national vaccine coverage was determined to be 56.09% and 63.7% in 2001 and 2003 respectively [28]. Our results show that particularly in persons over 65 years of age, but also in those under 5, there were excess hospital admissions in periods of influenza A(H3N2) activity. These findings encourage the continued use and strengthening of campaigns to increase vaccination coverage in these age groups.

Data from the SISS, allowing the early identification of circulating virulent serotypes and the start of epidemic periods, could be used to plan hospital resources for expected increases in flu-associated admissions.

We also recommend that further studies try to quantify the hospitalisation costs and days lost of work associated to flu epidemics in order to evaluate what economical impact flu epidemics suppose in Spain. The development of time series models including variables such as temperature, humidity, age group, sex, flu and other winter virus' circulation, will allow a better understanding of the role that each of these factors play during flu epidemics with regards to flu-associated pathologies and their presence in hospitals.

## Conclusion

Flu epidemics have an impact on hospital morbidity in Spain. Further studies that include other variables, such as temperature and humidity are necessary and will deepen our understanding of the role of each factor during flu epidemics and their relation with morbidity.

## Competing interests

The author(s) declare that they have no competing interests.

## Authors' contributions

ADL, VH and PR were involved in designing the study and participated in the collection and analysis of the data. ADL drafted the manuscript and AL, JDMD and SDM provided important intellectual content to the study. All authors have read and approved the final manuscript.

## Pre-publication history

The pre-publication history for this paper can be accessed here:



## Supplementary Material

Additional file 1 All-cause influenza-associated and disease specific hospitalisations/100,000/week for epidemic and non-epidemic periods during influenza surveillance seasons 2001/2002, 2002/2003 and 2003/2004 in Spain. Public hospital discharge registers and incidence of influenza-like-illness and viral isolation during flu seasons. Click here for file

Additional file 2 Excess all-cause influenza-associated and disease specific hospitalisations/100,000/week in persons < 5 years and over 64 years of age during flu epidemics the 2001/2002, 2002/2003 and 2003/2004 seasons in Spain. Public hospital discharge registers and incidence of influenza-like-illness and viral isolation during flu seasons. Click here for file
