# Supplementary material for: Impact of flu on hospital admissions during 4 flu seasons in Spain, 2000–2004
Source: BMC Public Health. 2007 Aug 8;7:197. doi: 10.1186/1471-2458-7-197 (PMC1964764; doi:10.1186/1471-2458-7-197)
Supplement: Additional file 1 — All-cause influenza-associated and disease specific hospitalisations/100,000/week for epidemic and non-epidemic periods during influenza surveillance seasons 2001/2002, 2002/2003 and 2003/2004 in Spain. Public hospital discharge registers and incidence of influenza-like-illness and viral isolation during flu seasons. [file 1471-2458-7-197-S1.doc]

**Table 1 - All-cause influenza-associated and disease specific hospitalisations/100,000/week** for epidemic and non-epidemic periods during influenza surveillance seasons 2001/2002, 2002/2003 and 2003/2004 in Spain.

| **Season** | **Type/ dominant subtype** | **Period** | **Duration (weeks)** | All Cause * **(CI95%)** | **Pneumonia***  **(CI95%)** | **Chronic Bronchitis***  **(CI95%)** | **Heart Failure***  **(CI95%)** | **Influenza***  **(CI95%)** |
| --- | --- | --- | --- | --- | --- | --- | --- | --- |
| **2001/2002 (S2)** | A(H3N2)/B | Epidemic | 12 | 9.64  (9.63-9.65) | 7.77  (7.76-7.78) | 2.21  (2.21-2.22) | 1.61  (1.61-1.61) | 0.06  (0.06-0.06) |
|  |  | Non-epidemic | 21 | 6.25  (6.24-6.25) | 4.94  (4.93-4.94) | 1.43  (1.42-1.43) | 1.11  (1.11-1.12) | 0.01  (0.01-0.01) |
|  |  | Excess |  | 3.40  (3.39-3.40) | 2.83  (2.82-2.84) | 0.79  (0.78-0.79) | 0.50  (0.49-0.50) | 0.05  (0.05-0.06) |
| **2002/2003 (S3)** | B/A(H1N1) | Epidemic | 14 | 5.28  (5.27-5.29) | 4.84  (4.84-4.85) | 0.89  (0.89-0.89) | 0.69  (0.69-0.69) | 0.05  (0.05-0.05) |
|  |  | Non-epidemic | 19 | 5.57  (5.56-5.58) | 4.64  (4.63-4.65) | 1.14  (1.14-1.14) | 0.86  (0.86-0.87) | 0.02  (0.02-0.02) |
|  |  | Excess |  | -0.29  (-0.29- -0.29) | 0.21  (0.20-0.21) | -0.25  (-0.25- -0.25) | -0.17  (-0.17- -0.17) | 0.03  (0.03-0.04) |
| **2003/2004 (S4)** | A(H3N2)/B | Epidemic | 10 | 5.95  (5.94-5,96) | 5.37  (5.37-5.38) | 0.96  (0.96-0.97) | 0.73  (0.72-0.73) | 0.15  (0.14-0.15) |
|  |  | Non-epidemic | 23 | 5.09  (5.08-5.10) | 4.68  (4.67-4.68) | 0.86  (0.86-0.86) | 0.68  (0.68-0.68) | 0.02  (0.02-0.03) |
|  |  | Excess |  | 0.86  (0.86-0.86) | 0.70  (0.69-0.70) | 0.10  (0.10-0.10) | 0.05  (0.04-0.05) | 0.12  (0.12-0.12) |
