# Supplementary material for: Impact of flu on hospital admissions during 4 flu seasons in Spain, 2000–2004
Source: BMC Public Health. 2007 Aug 8;7:197. doi: 10.1186/1471-2458-7-197 (PMC1964764; doi:10.1186/1471-2458-7-197)
Supplement: Additional file 2 — Excess all-cause influenza-associated and disease specific hospitalisations/100,000/week in persons < 5 years and over 64 years of age during flu epidemics the 2001/2002, 2002/2003 and 2003/2004 seasons in Spain. Public hospital discharge registers and incidence of influenza-like-illness and viral isolation during flu seasons. [file 1471-2458-7-197-S2.doc]

**Table 2: Excess all-cause influenza-associated and disease specific hospitalisations/100,000/week in persons < 5 years and over 64 years of age during flu epidemics the 2001/2002, 2002/2003 and 2003/2004 seasons in Spain.**

| **Age group** | **Season** | Excess | | | | |
| --- | --- | --- | --- | --- | --- | --- |
|  |  | All Cause * **(CI95%)** | **Pneumonia***  **(CI95%)** | **Chronic Bronchitis***  **(CI95%)** | **Heart Failure***  **(CI95%)** | **Influenza***  **(CI95%)** |
|  | **2001/2002** | 10.53  (10.49-10.57) | 10.32  (10.28-10.36) | 0.34  (0.33-0.34) | 0.03  (0.02-0.03) | 0.27  (0.26-0.27) |
| **Under 5** | **2002/2003** | 0.37  (0.36-0.37) | 0.19  (0.19-0.20) | -0.05  (-0.05- -0.05) | 0.00  (0.00-0.00) | 0.19  (0.19-0.20) |
|  | **2003/2004** | 10.17  (10.13-10.21) | 10.09  (10.05-10.13) | 0.13  (0.12-0.13) | 0.08  (0.07-0.08) | 0.90  (0.89-0.92) |
|  | **2001/2002** | 12.21  (12,19-12,24) | 9.76  (9.74-9.78) | 3.54  (3.53-3.56) | 2.53  (2.51-2.54) | 0.11  (0.11-0.11) |
| **Over 64** | **2002/2003** | -1.10  (-1.11- .1.09) | 1.24  (1.24-1.25) | -0.99  (-1.00- -0.98) | -0.75  (-0.76- -0.74) | 0.02  (0.02-0.02) |
|  | **2003/2004** | 14.19  (14.17-14.22) | 11.20  (11.18-11.23) | 2.77  (2.75-2.78) | 2.40  (2.39-2.41) | 0.03  (0.03-0.03) |

* Hospitalisations are calculated as the number of hospitalisations/population of the second year of the influenza season
